# Supplementary material for: DipM controls multiple autolysins and mediates a regulatory feedback loop promoting cell constriction in Caulobacter crescentus
Source: Nat Commun. 2023 Jul 11;14:4095. doi: 10.1038/s41467-023-39783-w (PMC10336132; doi:10.1038/s41467-023-39783-w)
Supplement: Supplementary file 1 — Supplementary Information [file 41467_2023_39783_MOESM1_ESM.pdf]

Supplementary Information

DipM controls multiple autolysins and mediates a regulatory feedback loop promoting cell constriction in *Caulobacter crescentus*

Izquierdo-Martinez A, Billini M, Miguel-Ruano V, Hernández-Tamayo R, Richter P, Biboy J, Batuecas MT, Glatter T, Vollmer W, Graumann PL, Hermoso JA, Thanbichler M

Supplementary figures

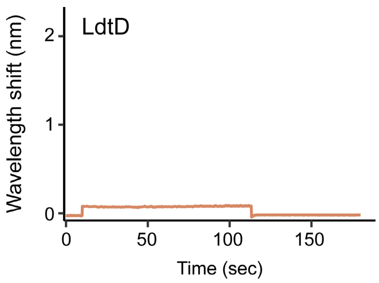

Supplementary Figure 1. Lack of interaction of DipM with the L,D-transpeptidase LdtD from *C. crescentus*. Biotinylated LdtD was immobilized on a BLI sensor and probed with DipM (20 µM). The graph shows a representative experiment (n=2 independent replicates).

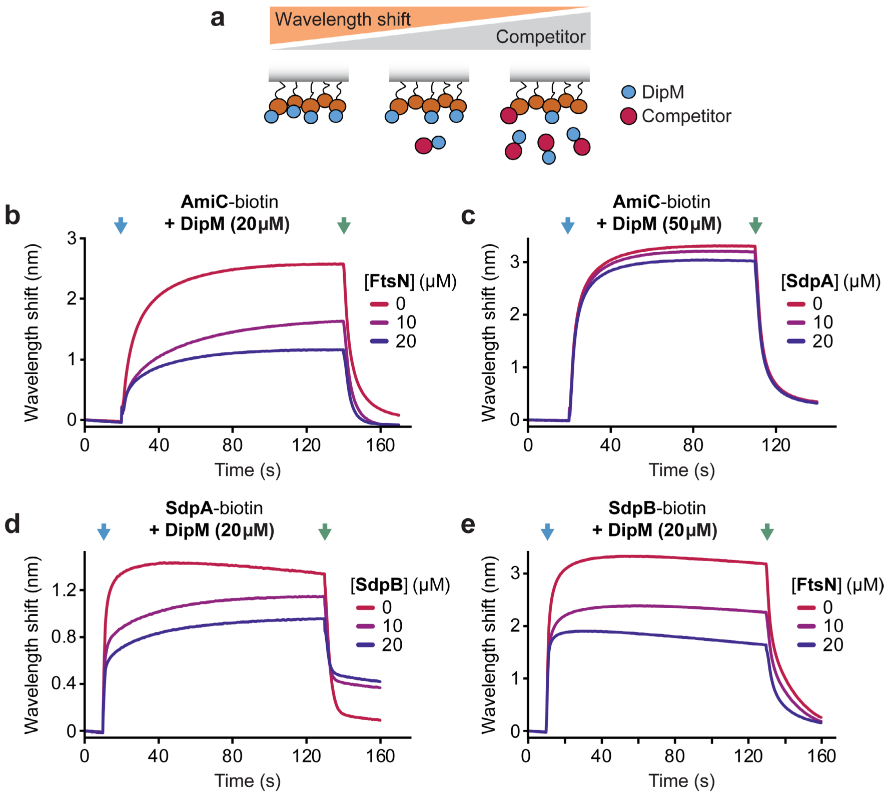

Supplementary Figure 2. The different interactors compete for binding to DipM. (a) Schematic representation of the BLI-based competition assay used in this study. After immobilization of one of the interactors on the sensor surface, the sensor is probed with DipM alone or with mixtures of DipM and a second interactor. If the two interactors bind to different, non-overlapping sites on DipM, they form a ternary complex on the sensor, leading to an increase in the wavelength shift. Otherwise, the signal remains largely constant or decreases with increasing concentrations of the second interactor, depending on the relative affinities of the interactions. **(b-e)** Competitive binding of two interactors to DipM. Sensors derivatized with the indicated biotinylated interactor were probed with DipM alone or with a mixture of DipM preincubated with a second interactor at the indicated concentrations. A blue arrow marks the start of the association phase, a green arrow the start of the dissociation phase. All assays were performed at least in duplicate, with similar results obtained throughout.

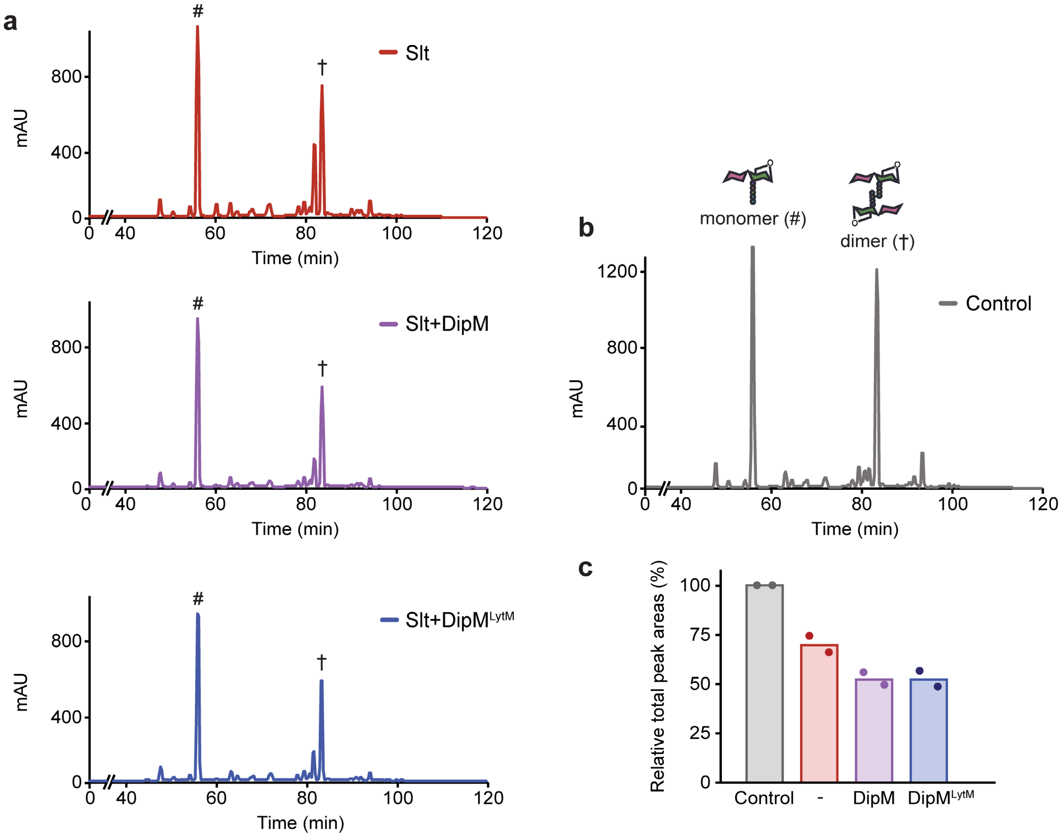

Supplementary Figure 3. Effect of DipM or DipM^{LytM} on the activity of the soluble lytic transglycosylase Slt from *E. coli*. (a) Slt (5 µM) was incubated with murein sacculi alone or with an equimolar amount of DipM or DipM^{LytM} and incubated for 30 min. Subsequently, the muropeptides generated by separated by HPLC and identified based on the elution times of reference compounds. Hash signs (#) mark the peaks of monomeric products, daggers (†) those of dimeric products. **(b)** Slt (5 µM) was incubated with murein sacculi in the absence of additional proteins for 2 h prior to muropeptide analysis in order to determine the maximal amount of product that can be achieved. **(c)** The graphs show the total amount of all muropeptide species obtained in the reactions described in panels a and b (n=2 independent experiments). Source data are provided as a Source Data file.

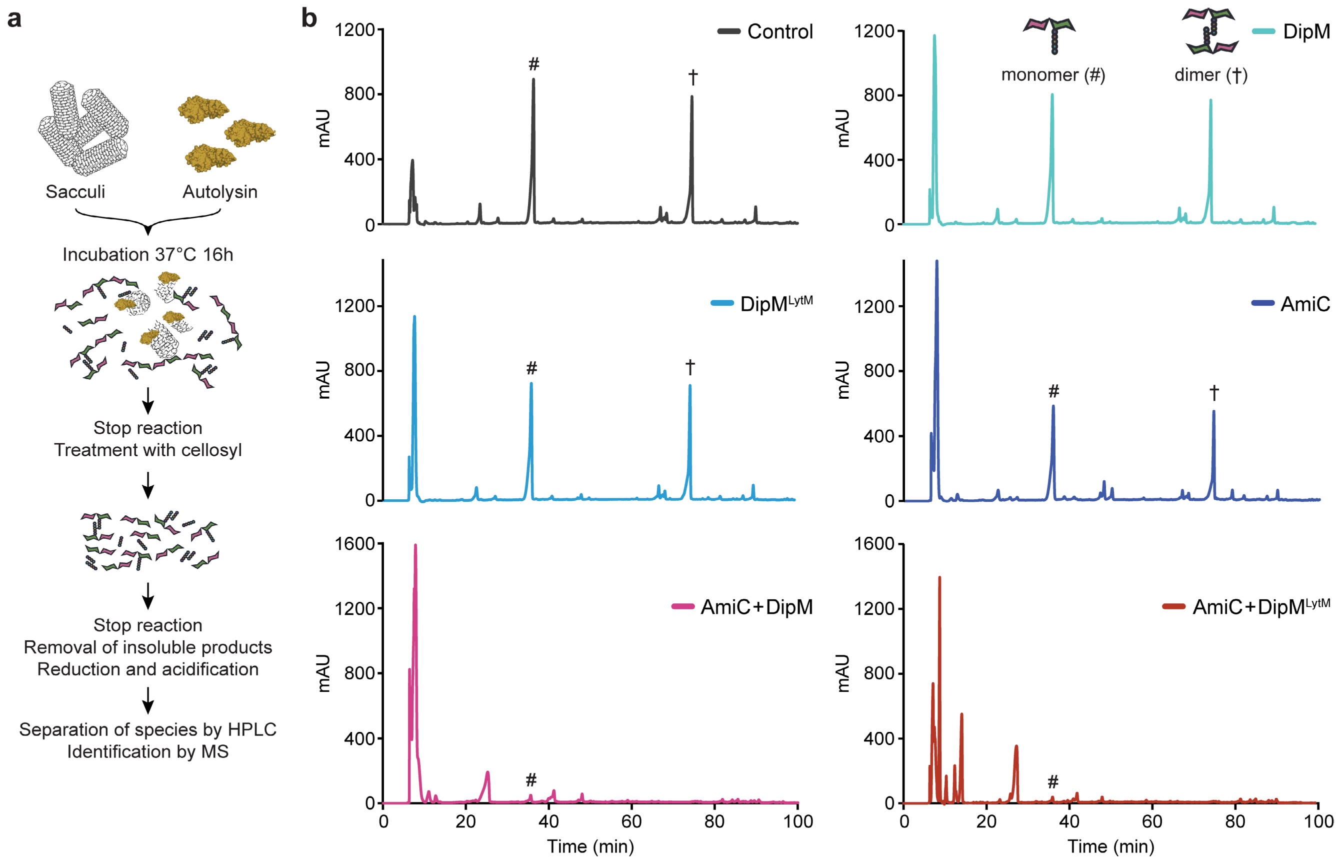

Supplementary Figure 4. DipM-dependent stimulation of the amidase activity of AmiC. (a) Overview of the procedure used to assess the activity of AmiC. **(b)** HPLC chromatograms showing the muropeptides generated by the treatment of sacculi with cellosyl (Control) and changes in the muropeptide profile resulting from the subsequent incubation of these muropeptides with the indicated protein(s). AmiC and DipM/DipM^{LytM} were used at equimolar ratios. Hash signs (#) mark the peaks of monomeric products, daggers (†) those of dimeric products. Amidase activity is indicated by a decrease in the abundance of these species, because the reaction generates free sugar dimers and peptides, which do not bind to the column under the conditions used.

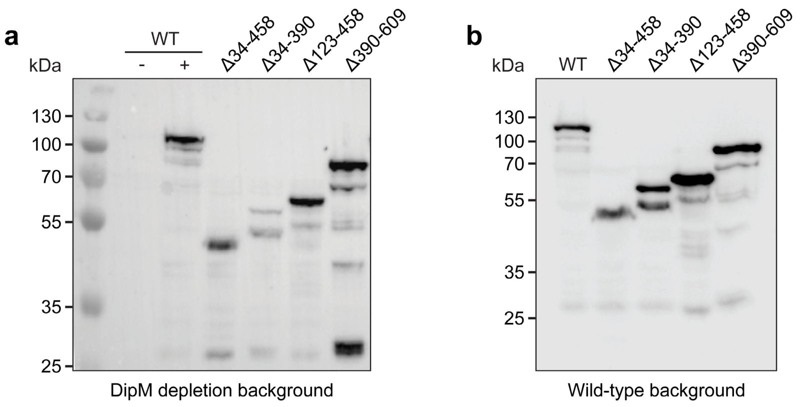

Supplementary Figure 5. Immunoblot blot analysis of strains producing mutant DipM-msfTurquoise2[ox] variants. (a) Strains producing the native DipM protein under the control of a xylose-inducible promoter and the indicated DipM-sfmTurquoise2[ox] variants under the control of a vanillate-inducible promoter (MAB512, MAB501, MAB502, MAB503, MAB513) were grown in PYE medium containing xylose and diluted into PYE medium containing vanillate to deplete DipM and induce the respective DipM-sfmTurquoise2[ox] fusion. The cells were then incubated for another 18 h prior to immunoblot analysis with an anti-GFP antibody (which also recognizes sfmTurquoise2[ox]). A culture (MAB512) grown in medium lacking vanillate (-) was analyzed as a control. **(b)** Immunoblot analysis of the strains described in panel a, grown in PYE medium containing xylose and induced for 3 h with vanillate prior to harvest. The indicated DipM-sfmTurquoise2[ox] fusions are produced in the presence of the native DipM protein, so that the cells still show wild-type morphology. The Western blot analyses were conducted at least twice, with similar results.

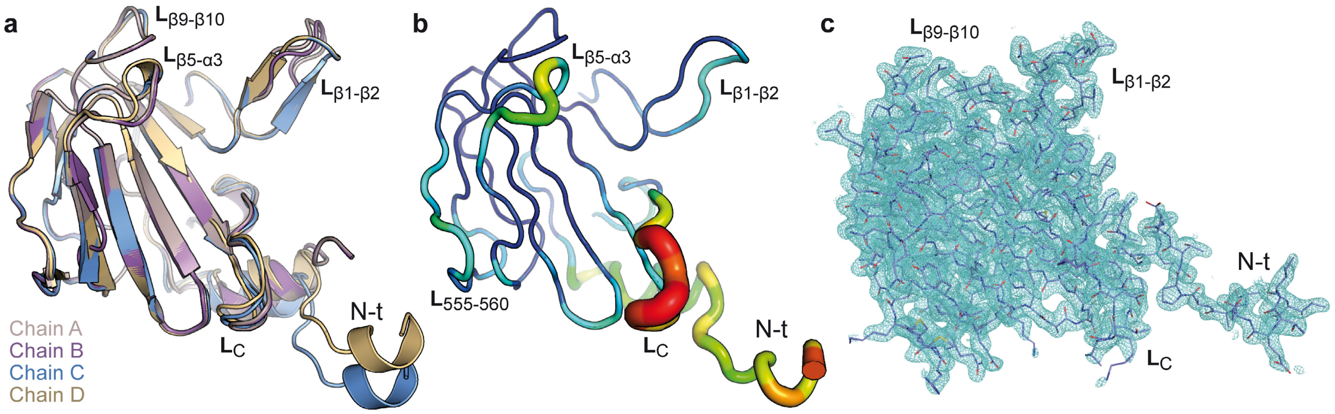

Supplementary Figure 6. Electron density, structural plasticity and B-factor distribution for the DipM[LytM] monomer. (a) Superimposition of the four independent molecules (chains A-D) constituting the asymmetric unit. Monomers are shown in cartoon view and colored differently. Variable regions are labeled. **(b)** Putty tube representation of the B-factor for the DipM[LytM] reference chain (chain C). The color of the backbone varies depending on the B-factor of the residues, ranging from blue (lowest) to red (highest). In addition, the diameter of the tube increases with the size of the B-factor. **(c)** 2Fo-Fc electron density map for DipM[LytM] chain C contoured at 1σ (shown as a blue mesh). Relevant regions are labeled.

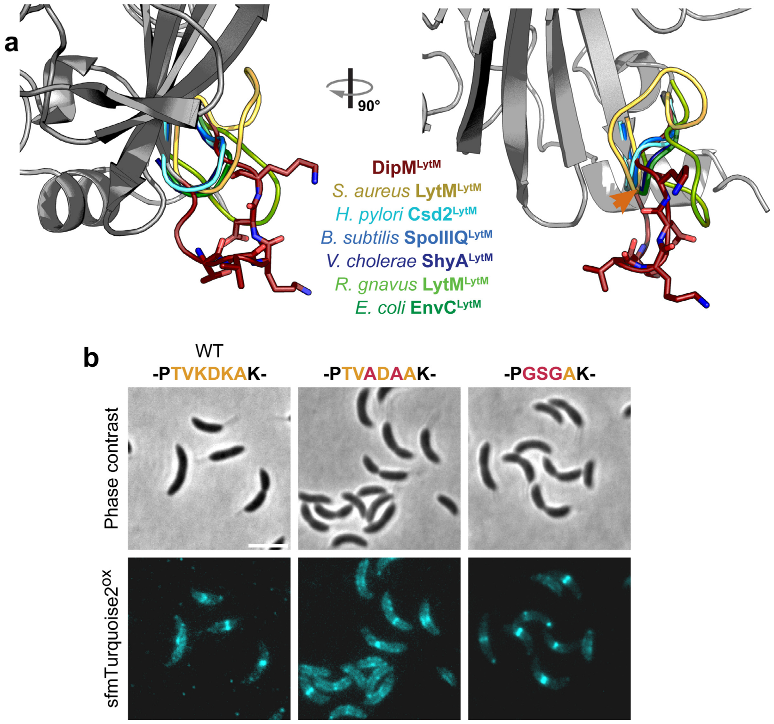

Supplementary Figure 7. Role of the DipM-specific loop at the C-terminal end of DipM^{LytM}. (a) Structural alignment of the crystal structures of DipM^{LytM} and the LytM domains of six other proteins, including *S. aureus* LytM (PDB: 4ZYB) (Grabowska et al., 2015), *H. pylori* Csd2 (PDB: 5J1L) (An et al., 2016), *B. subtilis* SpoIIIQ (PDB: 3UZ0) (Meisner et al., 2012), *V. cholerae* ShyA (PDB: 6UE4) (Shin et al., 2020), *R. gnavus* LytM (PDB: 3NYY) and *E. coli* EnvC (PDB: 4EH5). For all proteins except for DipM, only the loop following the last β-sheet of each LytM domain is shown for clarity, represented as colored ribbons without any secondary structural elements. The residues in the KDK motif, which is conserved in *C. crescentus* and close relatives, are shown in stick representation. The orange arrowhead indicates the position at which, in most structures, the loop turns upwards. **(b)** Functionality of DipM variants with exchanges in the conserved KDKA motif. Shown are phase contrast and fluorescence images of cells producing the indicated DipM-sfmTurquoise2^{ox} variants in place of the native protein (MAB512, MAB515, MAB514) in 2xPYE medium (scale bar: 3 µm). The native sequence of the conserved loop (orange) and residues exchanged in the mutant variants (red) are given on top of the corresponding images. All microscopic analyses were performed twice, with similar results.

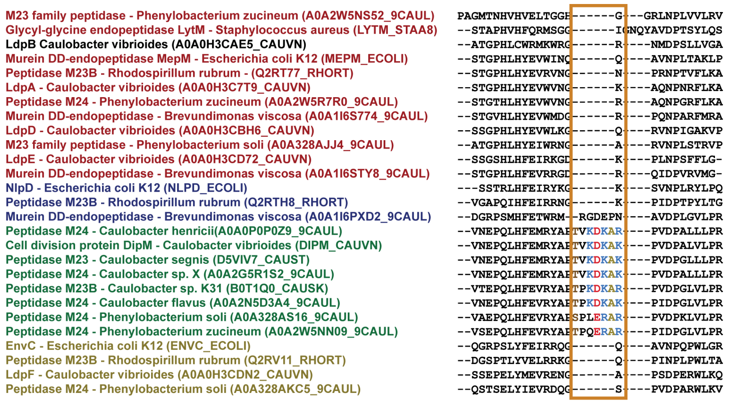

Supplementary Figure 8. Alignment of the C-terminal regions of DipM and other LytM domain-containing proteins. DipM homologs of the genera *Caulobacter* and *Phenylobacterium* are shown in green, NlpD homologs of alpha- and gammaproteobacteria in blue, EnvC homologs of alpha- and gammaproteobacteria in olive, and catalytically active LytM domain-containing proteins from alpha- and gammaproteobacteria as well as LytM of *S. aureus* in red. *C. crescentus* LdpB, whose catalytic proficiency is still unclear, is shown in black. The conserved loop present in the *Caulobacter* and *Phenylobacterium* homologs is highlighted with an orange box and its conserved residues are shown in color.

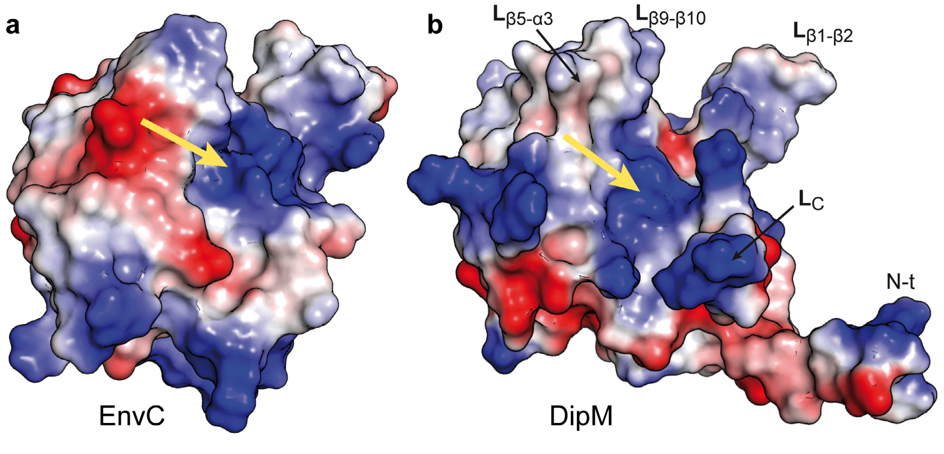

Supplementary Figure 9. Positive electrostatic surface potential of the putative AmiC binding groove of DipM^{LytM}. Shown is the electrostatic surface potential of **(a)** the LytM domain of EnvC and **(b)** DipM^{LytM}, with positive and negative charges colored in blue and red, respectively. Yellow arrows point to the positive electrostatic potential in the binding groove. Loops delimiting the cavity in DipM are labeled.

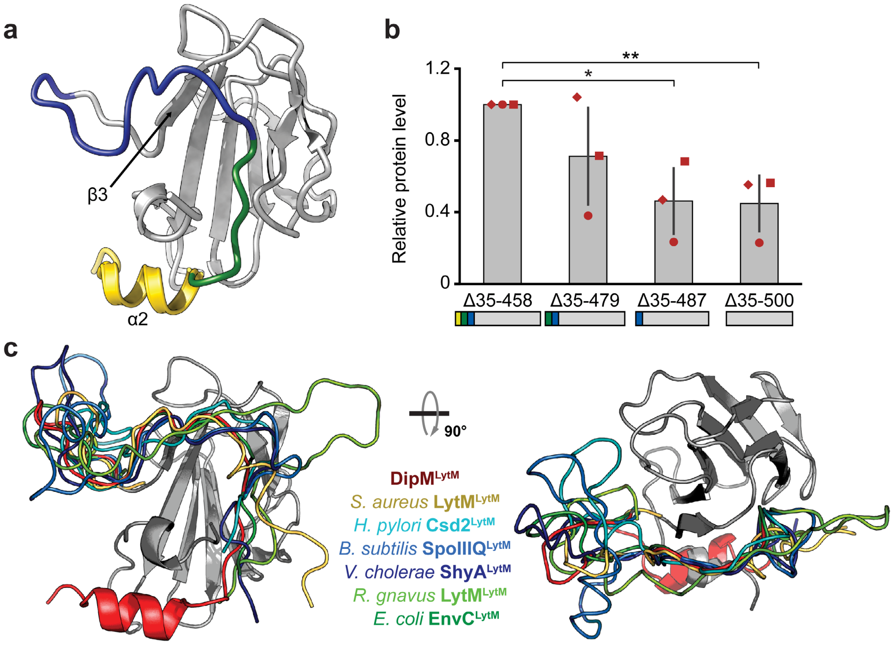

Supplementary Figure 10. Requirement of the N-terminal region of DipM^{LytM} for protein stability. **(a)** Shown is a cartoon representation of DipM^{LytM}. The part of the protein that is recognized by the Hidden Markov Model employed to identify the LytM domain by the Pfam database (Mistry et al., 2021) is shown in grey. The remaining N-terminal region is divided into three parts: residues that are closer to the LytM domain and contact strand β2 and adjacent regions (blue), the following segment up to helix α2 (green) and helix α2 (yellow). **(b)** Bar chart representing the average (± SD) levels of the indicated DipM-sfmTurquoise2^{ox} variants (n=3 independent experiments), as determined by Western blot analysis of cells producing the fusion proteins in the wild-type background (AI098, AI123, AI124, AI125). The individual data points from the three replicates are shown as red symbols. The schematics at the bottom of the chart depict the architecture of the different protein variants. Asterisks indicate the statistical significance of differences between the averages obtained, determined by a one-way ANOVA (***** p=0.0143 and ****** p=0.0073). **(c)** Structural alignment of the crystal structures of DipM^{LytM} (in grey) and the LytM domains of six other proteins: *S. aureus* LytM (PDB: 4ZYB) (Grabowska et al., 2015), *H. pylori* Csd2 (PDB: 5J1L) (An et al., 2016), *B. subtilis* SpoIIIQ (PDB: 3UZ0) (Meisner et al., 2012), *V. cholerae* ShyA (PDB: 6UE4) (Shin et al., 2020), *R. gnavus* LytM (PDB: 3NYY) and *E. coli* EnvC (PDB: 4EH5). For all proteins except for DipM, only the N-terminal region adjacent to the LytM domain is shown for clarity, represented as colored ribbons without any secondary structural elements. Source data are provided as a Source Data file.

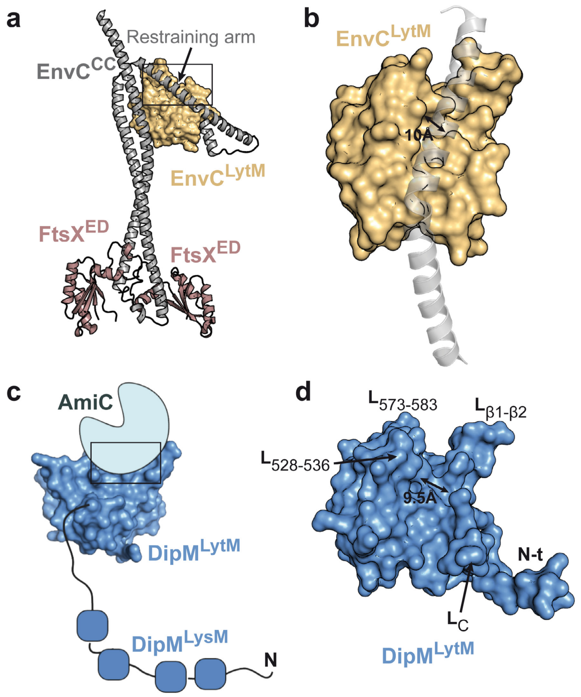

Supplementary Figure 11. Comparison of the structures of EnvC and DipM. (a) Crystal structure of EnvC bound to the periplasmic domain of FtsX ($FtsX^{ED}$). The LytM domain of EnvC ($EnvC^{LytM}$) and its N-terminal coiled-coil region ($EnvC^{CC}$) are indicated (PDB: 6TPI) (Cook et al., 2020). **(b)** Detailed view of the self-inhibitory structure form through interaction of $EnvC^{LytM}$ (yellow) with the restraining arm (transparent gray cartoon), highlighted by a black box in panel A. **(c)** Schematic model of the $DipM^{LytM}$-AmiC complex of *C. crescentus*, as predicted by AlphaFold-Multimer (Evans et al., 2022) (detailed in Figure 6c). **(d)** Surface view of $DipM^{LytM}$, arranged in the same orientation as EnvC in panel b.

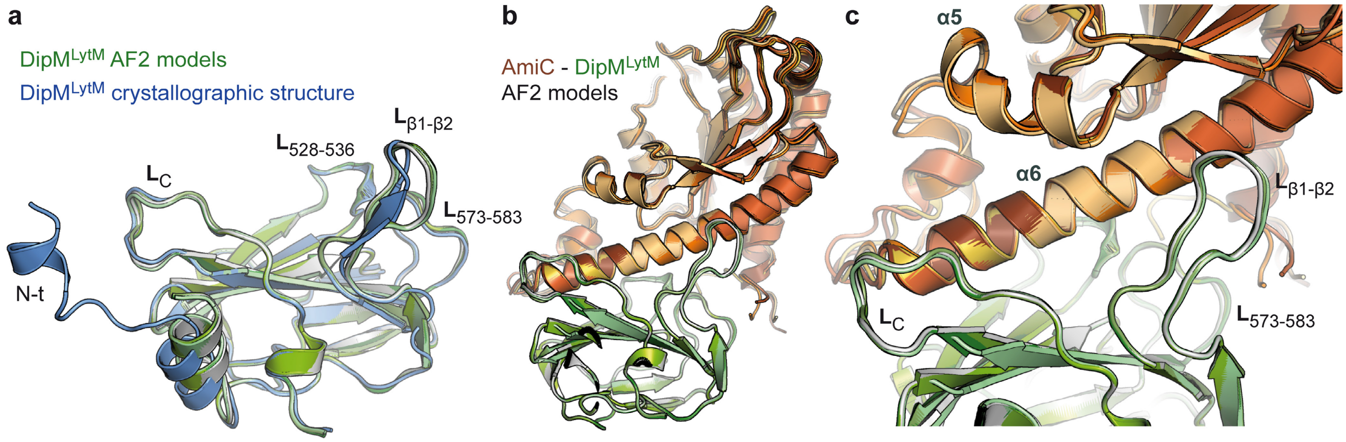

Supplementary Figure 12. Evaluation of the model of the DipM^{LytM}-AmiC complex generated by AlphaFold-Multimer. (a) Structural superimposition of the crystal structure of DipM^{LytM} (chain C, in blue) and a model of DipM^{LytM} generated by AlphaFold-Multimer (Evans et al., 2022) (in various shades of green). **(b)** Superimposition of DipM^{LytM}-AmiC complexes predicted by AlphaFold-Multimer. DipM^{LytM} is shown in green, the different AmiC models in various shades of orange. **(c)** Magnified view of the predicted interacting regions of Dip^{LytM} and AmiC.

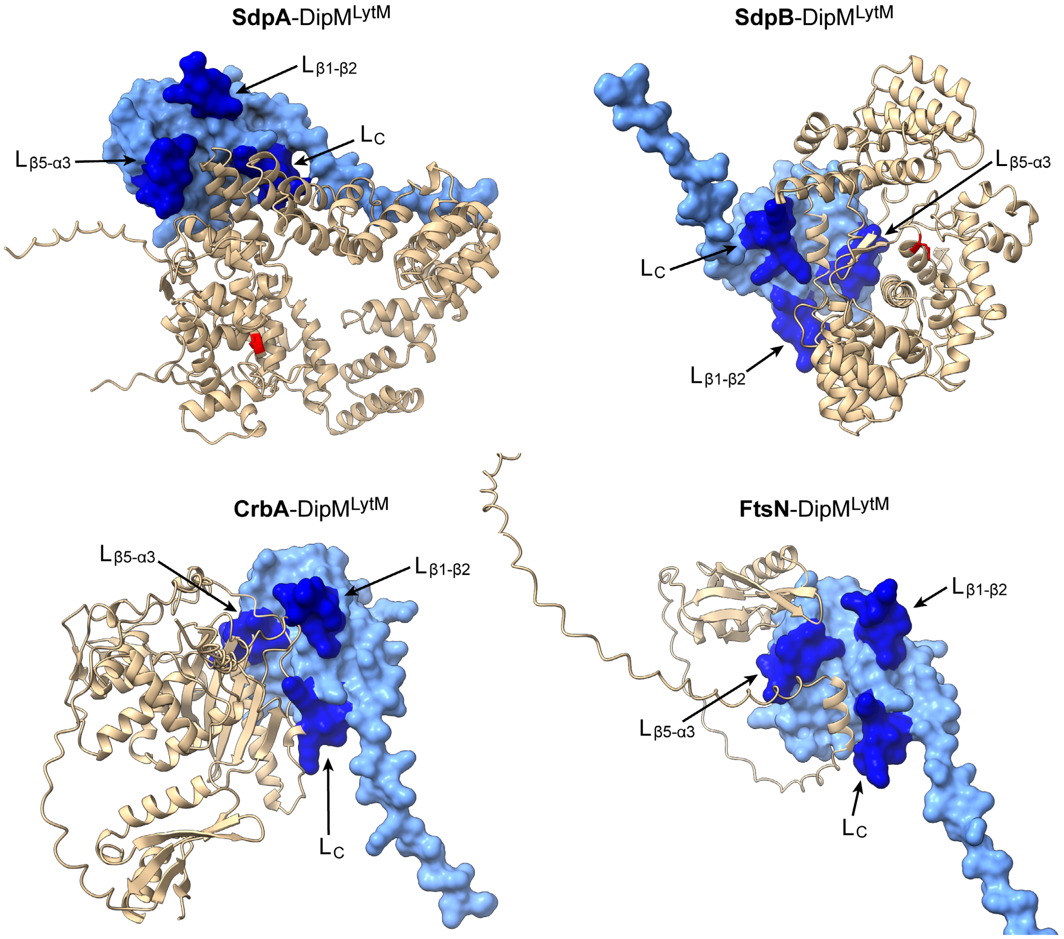

Supplementary Figure 13. Models of the complexes formed by DipM^{LytM} with SdpA, SdpB, CrbA and FtsN. Shown are the top-ranking models of the indicated DipM^{LytM}-interactor complexes generated by AlphaFold-Multimer (Evans et al., 2022).

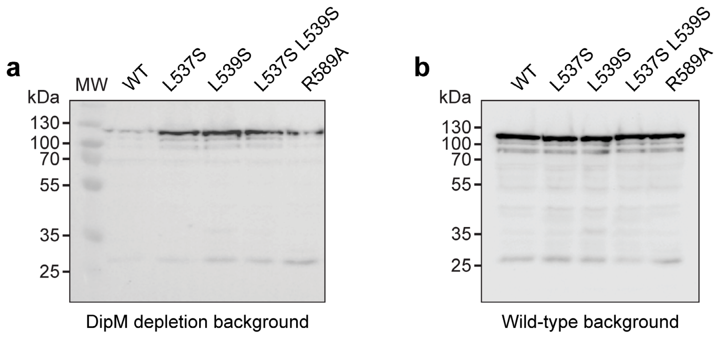

Supplementary Figure 14. Immunoblot blot analysis of strains producing DipM-msfTurquoise2^{ox} variants with exchanges in the LytM domain. (a) Strains producing the native DipM protein under the control of a xylose-inducible promoter and the indicated DipM-sfmTurquoise2^{ox} variants under the control of a vanillate-inducible promoter (MAB512, MAB505, MAB506, MAB510, MAB504) were grown in PYE medium containing xylose and diluted into PYE medium containing vanillate to deplete DipM and induce the respective DipM-sfmTurquoise2^{ox} fusion. The cells were then incubated for another 18 h prior to immunoblot analysis with an anti-GFP antibody (which also recognizes sfmTurquoise2^{ox}). **(b)** Immunoblot blot analysis of the strains described in panel a, grown in PYE medium containing xylose and induced with vanillate for 3 h prior to harvest. The cells produce the indicated DipM-sfmTurquoise2^{ox} fusions in addition to the native DipM protein and thus still show wild-type morphology. Under this condition, all fusion proteins accumulate to the same level, indicating that the mutations do not have any adverse effect on protein synthesis or stability. The Western blot analyses were conducted at least twice, with similar results.

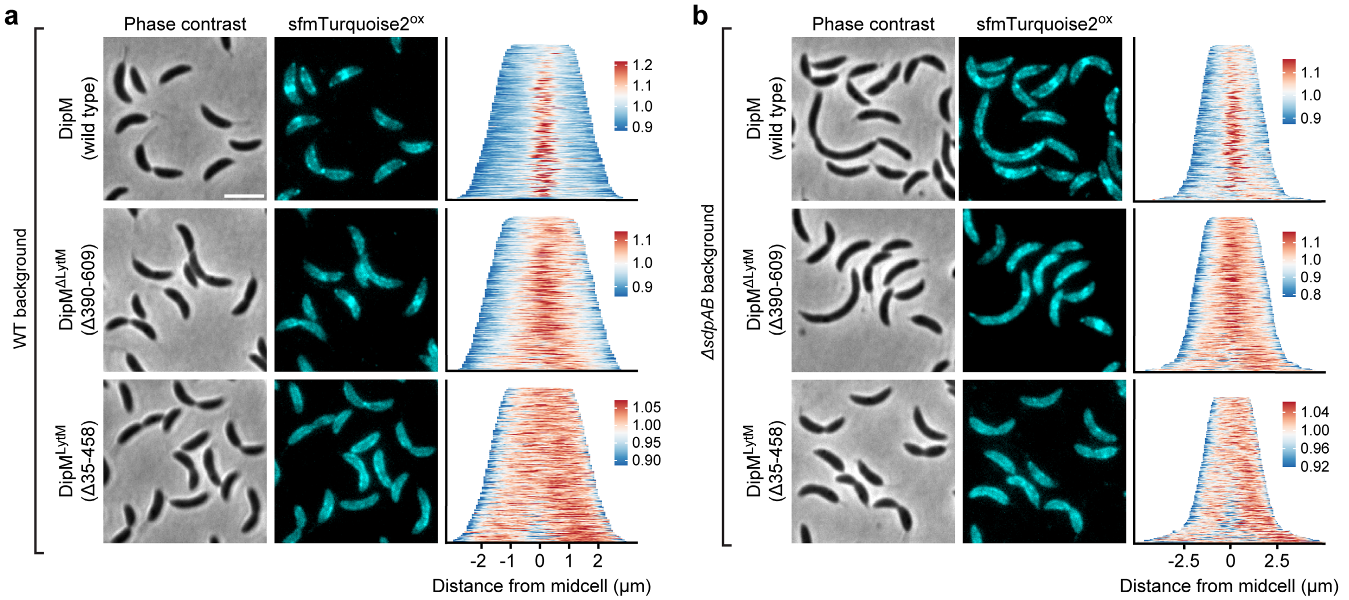

Supplementary Figure 15. Localization patterns of different DipM-sfmTurquoise2^{ox} variants in the wild-type and Δ*sdpAB* backgrounds. (a) Wild-type or **(b)** Δ*sdpAB* mutant cells carrying genes for the indicated DipM-sfmTurquoise2^{ox} fusions under the control of a xylose-inducible promoter (AI063, AI112, AI098, AI121, AI126, AI122) were induced with xylose for 3 h prior to analysis by phase contrast and fluorescence microscopy (scale bar: 3 µm). The demographs next to the microscopy images show the fluorescence profiles of representative subpopulations of cells (n=250) stacked on top of each other according to cell length. Source data are provided as a Source Data file.

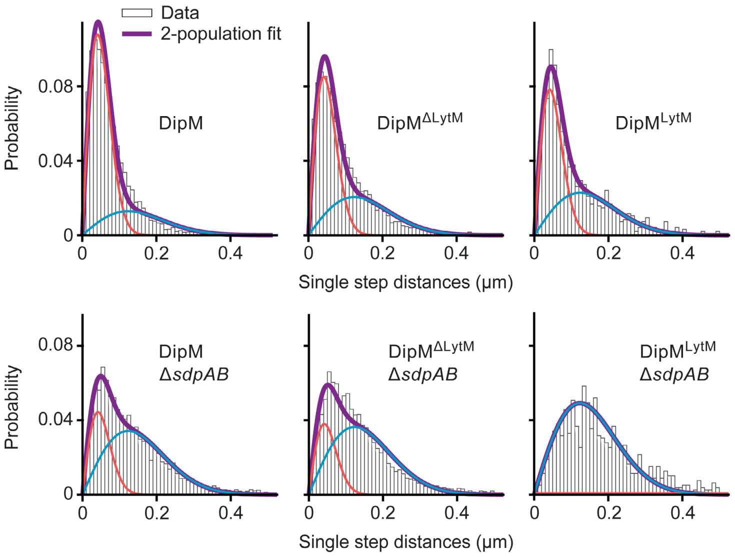

Supplementary Figure 16. **Single-molecule mobilities of different DipM variants.** Shown is a Gaussian-mixture-analysis of the mobility of the indicated DipM-sfmTurquoise2^{ox} variants (measured by single-particle tracking as described in the legend to Figure 9). The probability distributions of the single-step frame-to-frame displacements obtained in the single-particle tracking experiments were fitted to a two-component Gaussian function, assuming a slow-moving (red line) and fast-moving (blue line) population.

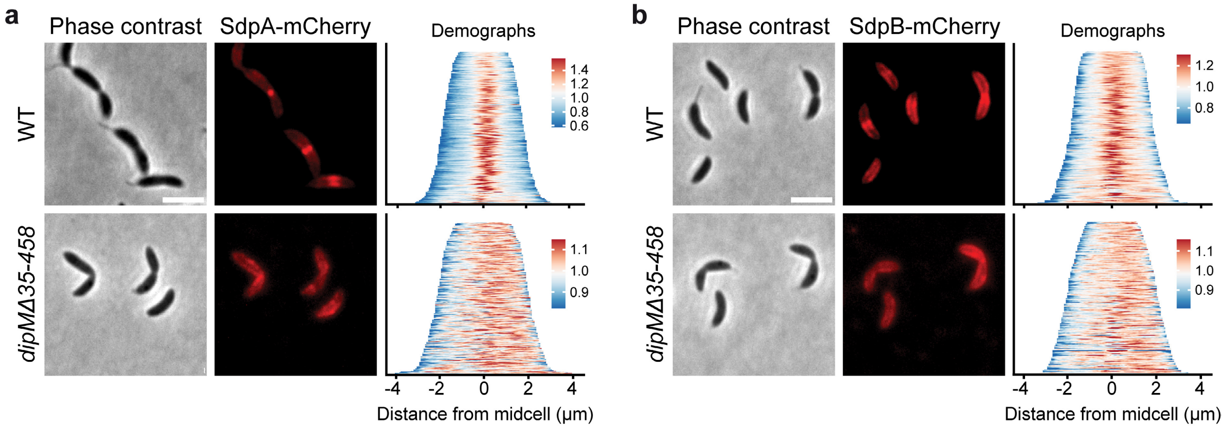

Supplementary Figure 17. **Impaired localization of SdpA-mCherry and SdpB-mCherry in cells producing DipM^{LytM} in place of the native DipM protein.** Wild-type (WT) or *dipMΔ35-458* cells carrying the gene for **(a)** SdpA-mCherry or **(b)** SdpB-mCherry under the control of a xylose-inducible promoter (AM408, AZ127, AI113, AI114) were induced with xylose for 3 h prior to analysis by phase contrast and fluorescence microscopy (scale bar: 3 µm). The demographs next to the microscopy images show the fluorescence profiles of representative subpopulations of cells (n>190) stacked on top of each other according to cell length. Only cells with a length similar to that of wild-type cells were included in the analysis. Source data are provided as a Source Data file.

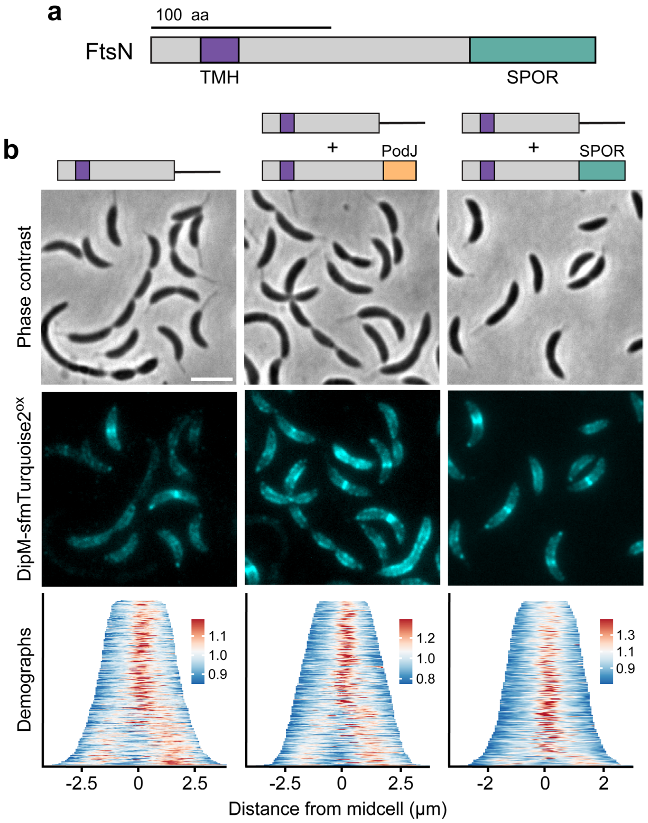

Supplementary Figure 18. Localization of DipM-sfmTurquoise2^{ox} in different *ftsN* mutant backgrounds. (a) Domain architecture of FtsN. **(b)** Phenotypes of cells producing different FtsN variants. A vanillate-inducible DipM-sfmTurquoise2^{ox} fusion was produced in cells whose native *ftsN* gene had been replaced by an allele encoding a truncated FtsN variant lacking the SPOR domain (AI117), which no longer condenses at the cell division site during cell constriction (Möll & Thanbichler, 2007). The same analysis was performed in strains that additionally produced an FtsN variant whose SPOR domain was replaced by a PG_binding_2 domain, which does not accumulate at midcell (Möll & Thanbichler, 2007) (MAB496), or by the wild-type FtsN protein (MAB494), each expressed at basal levels under the control of a xylose-inducible promoter (scale bar: 3 µm). The demographs show the fluorescence profiles of representative subpopulations of cells (n>200) stacked on top of each other according to cell length. Only non-chained cells were included in the analysis. Source data are provided as a Source Data file.

Supplementary tables

Supplementary Table 1. Data collection and refinement statistics for DipM^{LytM}.

Data Collection*	
Space group	$P\ 2\ 2_1\ 2_1$
Cell dimensions	
a, b, c (Å)	65.87, 105.84, 108.43
α, β, γ (deg)	90, 90, 90
Wavelength (Å)	0.979
Resolution (Å)	49.70- 2.25 (2.32-2.25)
R_{merge}	0.324 (1.512)
R_{pim}	0.102 (0.474)
$CC_{1/2}$	0.990 (0.703)
Mean $I/\sigma I$	8.0 (2.1)
Completeness (%)	100 (100)
Multiplicity	11.1 (11.1)
**Refinement**	
Resolution (Å)	49.75-2.25 (2.31-2.25)
Unique reflections	36758 (3319)
R_{work}/R_{free}	0.188/ 0.219
No. of atoms	
Non-hydrogen atoms	4902
Protein	4431
Ligands	0
Ions	0
Solvent	471
Ramachandran favored (%)	95.85
Ramachandran outliers (%)	0.00
Average B, all atoms (Å^2)	25.00
Root-mean-square deviation	
Bond lengths (Å)	0.010
Bond angles (deg)	1.569
Protein Data Bank entry	7QRL

*Values in parenthesis are for the highest-resolution shell.

Supplementary Table 2. Cells and tracks analyzed in the SMT analysis.

Strain	Cells	Tracks
WT - DipM	50	4572
WT - DipM$^{\Delta LytM}$	52	4213
WT - DipM$^{\Delta LytM}$	51	4323
Δ*sdpAB* - DipM	48	3976
Δ*sdpAB* - DipM$^{\Delta LytM}$	50	3365
Δ*sdpAB* - DipM$^{\Delta LytM}$	51	6067

Supplementary Table 3. Strains used in this study.

Strain name	Genotype	Construction/reference
***E. coli* strains**		
Rosetta(DE3) pLysS	F^- *ompT hsdS*$_B$(r_B^- m_B^-) *gal dcm* (DE3) pLysSRARE (Cam^R)	Merck Millipore
TOP10	cloning strain	Invitrogen
WM3064	*thrB*1004 *pro thi rpsL hsdS lacZ*ΔM15 RP4–1360 Δ(*araBAD)567* Δ*dapA*1341::[*erm pir*(wt)]	W. Metcalf (unpublished)
AI033	Rosetta(DE3)pLysS bearing a pET28(a)+ derivative encoding SdpA$_{(21\text{-}699)}$-His$_6$	Transformation of Rosetta(DE3)pLysS with pAI014
AI041	Rosetta(DE3)pLysS bearing a pTB146 derivative encoding His$_6$-SUMO-dipM$_{(459\text{-}609)}$	Transformation of Rosetta(DE3)pLysS with pAI001
AI046	Rosetta(DE3)pLysS bearing a pTB146 derivative encoding His$_6$-SUMO-SdpB$_{(26\text{-}536)}$	Transformation of Rosetta(DE3)pLysS with pAI026
AI060	Rosetta(DE3)pLysS bearing a pTB146 derivative encoding His$_6$-SUMO-FtsN$_{(51\text{-}266)}$	Transformation of Rosetta(DE3)pLysS with pAI036
AI061	Rosetta(DE3)pLysS bearing a pTB146 derivative encoding His$_6$-SUMO-AmiC$_{(35\text{-}395)}$	Transformation of Rosetta(DE3)pLysS with pAI037
AI062	Rosetta(DE3)pLysS bearing a pTB146 derivative encoding His$_6$-SUMO-LdpF$_{(25\text{-}351)}$	Transformation of Rosetta(DE3)pLysS with pAI025
AI075	Rosetta(DE3)pLysS bearing a pTB146 derivative encoding His$_6$-SUMO-CrbA$_{(371\text{-}451)}$	Transformation of Rosetta(DE3)pLysS with pAI049
AM201	Rosetta(DE3)pLysS bearing a pET28a(+) derivative encoding DipM$_{(26\text{-}610)}$-His$_6$	Möll et al., 2010
MAB408	Rosetta(DE3)pLysS bearing a pTB146 derivative encoding His$_6$-SUMO-LdtD$_{(26\text{-}502)}$	Transformation of Rosetta(DE3)pLysS with pMAB150
MAB493	Rosetta(DE3)pLysS bearing a pET28a(+) derivative encoding the L539S variant of DipM$_{(26\text{-}610)}$-His$_6$	Transformation of Rosetta(DE3)pLysS with pMAB206
MAB500	Rosetta(DE3)pLysS bearing a pET28a(+) derivative encoding the L537S L539S variant of DipM$_{(26\text{-}610)}$-His$_6$	Transformation of Rosetta(DE3)pLysS with pMAB207
MAB516	Rosetta(DE3)pLysS bearing a pET28a(+) derivative encoding the R589A variant of DipM$_{(26\text{-}610)}$-His$_6$	Transformation of Rosetta(DE3)pLysS with pMAB194
***C. crescentus* strains**		
CB15N	Synchronizable derivative of the wild-type strain CB15	Evinger and Agabian, 1977
AM369	CB15N Δ*ldpF*	Zielińska et al., 2017
AM376	CB15N Δ*crbA*	Billini et al., 2019
AM399	CB15N Δ*sdpA*	Zielińska et al., 2017
AM3	CB15N *ftsN*::*ftsN*Δ*SPOR*	Möll and Thanbichler, 2009
AM418	CB15N Δ*sdpB*	Zielińska et al., 2017
AM419	CB15N Δ*sdpA* Δ*sdpB*	Zielińska et al., 2017
MT46	CB15N *ftsN*::*ftsN-gfp*	Möll and Thanbichler, 2009
AI018	CB15N P_{xyl}::P_{xyl}-*dipM-flag*	Integration of pAI003 in CB15N
AI021	CB15N Δ*dipM* P_{xyl}::P_{xyl}-*dipM-flag*	In-frame deletion of *dipM* in AI018 using pMT814
AI032	CB15N Δ*sdpA* P_{xyl}::P_{xyl}-*sdpA-flag*	Integration of pAI013 in AM399
AI034	CB15N Δ*sdpA* P_{xyl}::P_{xyl}-*sdpA*	Integration of pAI015 in AM399
AI036	CB15N Δ*ldpF* P_{xyl}::P_{xyl}-*ldpF-flag*	Integration of pAI016 in AM369
AI038	CB15N Δ*crbA* P_{xyl}::P_{xyl}-*crbA-flag*	Integration of pAI018 in AM376
AI039	CB15N Δ*crbA* P_{xyl}::P_{xyl}-*crbA*	Integration of pAI019 in AM376
AI040	CB15N Δ*ldpF* P_{xyl}::P_{xyl}-*ldpF*	Integration of pAZ39 in AM369
AI052	CB15N P_{xyl}::P_{xyl}-*amiC-flag*	Integration of pAI029 in CB15N
AI053	CB15N Δ*amiC* P_{xyl}::P_{xyl}-*amiC-flag*	In-frame deletion of *amiC* in AI052 using pAM123
AI063	CB15N P_{xyl}::P_{xyl}-*dipM-sfmturquoise2*^{ox}	Integration of pAI039 in CB15N
AI097	CB15N *dipM*::*dipM*$_{(\Delta 36\text{-}458)}$	In-frame truncation of native *dipM* using pAI072
AI098	CB15N P_{xyl}::P_{xyl}-*dipM*$_{(\Delta 34\text{-}458)}$-*sfmturquoise2*^{ox}	Integration of pAI068 in CB15N
AI112	CB15N P_{xyl}::P_{xyl}-*dipM*$_{(\Delta 390\text{-}609)}$-*sfmturquoise2*^{ox}	Integration of pAI087 in CB15N
AI113	CB15N *dipM*::*dipM*$_{(\Delta 36\text{-}458)}$ P_{xyl}::P_{xyl}-*sdpA-mCherry*	Integration of pAM210 in AI097
AI114	CB15N *dipM*::*dipM*$_{(\Delta 36\text{-}458)}$ P_{xyl}::P_{xyl}-*sdpB-mCherry*	Integration of pAZ14 in AI097
AI117	CB15N *ftsN*::*ftsN*Δ*SPOR* P_{xyl}::P_{xyl}-*dipM-sfmturquoise2*^{ox}	Integration of pAI063 in AM3
AI121	CB15N Δ*sdpA* Δ*sdpB* P_{xyl}::P_{xyl}-*dipM-sfmturquoise2*^{ox}	Integration of pAI067 in AM419
AI122	CB15N Δ*sdpA* Δ*sdpB* P_{xyl}::P_{xyl}-*dipM*$_{(\Delta 34\text{-}458)}$-*sfmturquoise2*^{ox}	Integration of pAI068 in AM419

Supplementary Table 3. Strains used in this study (continued).

AI123	CB15N P_{xyl}::P_{xyl}-*dipM*$_{(\Delta 35\text{-}500)}$-*sfmturquoise2*^{ox}	Integration of pAI081 in CB15N
AI125	CB15N P_{xyl}::P_{xyl}-*dipM*$_{(\Delta 35\text{-}486)}$-*sfmturquoise2*^{ox}	Integration of pAI083 in CB15N
AI126	CB15N Δ*sdpA* Δ*sdpB* P_{xyl}::P_{xyl}-*dipM*$_{(\Delta 390\text{-}609)}$-*sfmturquoise2*^{ox}	Integration of pAI087 in AM419
MAB203	CB15N Δ*dipM* P_{xyl}::P_{xyl}-*sdpA-mcherry*	Zielińska et al., 2017
MAB308	CB15N Δ*dipM* P_{xyl}::P_{xyl}-*sdpB-mcherry*	Zielińska et al., 2017
MAB360	CB15N Δ*dipM* P_{xyl}::P_{xyl}-*dipM*	Zielińska et al., 2017
MAB386	CB15N Δ*amiC* P_{xyl}::P_{xyl}-*amiC*	Zielińska et al., 2017
MAB490	CB15N *ftsN*::*ftsN*Δ*SPOR* P_{xyl}::P_{xyl}-*venus-ftsN*	Integration of pAM14 in AM3
MAB492	CB15N *ftsN*::*ftsN*Δ*SPOR* P_{xyl}::P_{xyl}-*venus-ftsN*$_{(1\text{-}187)}$-*podJ*$_{(893\text{-}975)}$	Integration of pAM68 in AM3
MAB494	CB15N *ftsN*::*ftsN*Δ*SPOR* P_{xyl}::P_{xyl}-*venus-ftsN* P_{van}::P_{van}-*dipM-sfmturquoise2*^{ox}	Integration of pAI041 in MAB490
MAB496	CB15N *ftsN*::*ftsN*Δ*SPOR* P_{xyl}::P_{xyl}-*venus-ftsN*$_{(1\text{-}187)}$-*podJ*$_{(893\text{-}975)}$ P_{van}::P_{van}-*dipM-sfmturquoise2*^{ox}	Integration of pAI041 in MAB492
MAB501	CB15N Δ*dipM* P_{xyl}::P_{xyl}-*dipM* P_{van}::P_{van}-*dipM*$_{(\Delta 34\text{-}458)}$-*sfmturquoise2*^{ox}	Integration of pAI064 in MAB360
MAB502	CB15N Δ*dipM* P_{xyl}::P_{xyl}-*dipM* P_{van}::P_{van}-*dipM*$_{(\Delta 34\text{-}390)}$-*sfmturquoise2*^{ox}	Integration of pAI065 in MAB360
MAB503	CB15N Δ*dipM* P_{xyl}::P_{xyl}-*dipM* P_{van}::P_{van}-*dipM*$_{(\Delta 123\text{-}458)}$-*sfmturquoise2*^{ox}	Integration of pAI066 in MAB360
MAB504	CB15N Δ*dipM* P_{xyl}::P_{xyl}-*dipM* P_{van}::P_{van}-*dipM*$_{(R589A)}$-*sfmturquoise2*^{ox}	Integration of pAI095 in MAB360
MAB505	CB15N Δ*dipM* P_{xyl}::P_{xyl}-*dipM* P_{van}::P_{van}-*dipM*$_{(L537S)}$-*sfmturquoise2*^{ox}	Integration of pMAB195 in MAB360
MAB506	CB15N Δ*dipM* P_{xyl}::P_{xyl}-*dipM* P_{van}::P_{van}-*dipM*$_{(L539S)}$-*sfmturquoise2*^{ox}	Integration of pMAB198 in MAB360
MAB510	CB15N Δ*dipM* P_{xyl}::P_{xyl}-*dipM* P_{van}::P_{van}-*dipM*$_{(L537S\ L539S)}$-*sfmturquoise2*^{ox}	Integration of pMAB203 in MAB360
MAB512	CB15N Δ*dipM* P_{xyl}::P_{xyl}-*dipM* P_{van}::P_{van}-*dipM-sfmturquoise2*^{ox}	Integration of pAI063 in MAB360
MAB513	CB15N Δ*dipM* P_{xyl}::P_{xyl}-*dipM* P_{van}::P_{van}-*dipM*$_{(\Delta 390\text{-}609)}$-*sfmturquoise2*^{ox}	Integration of pAI075 in MAB360
MAB514	CB15N Δ*dipM* P_{xyl}::P_{xyl}-*dipM* P_{van}::P_{van}-*dipM*$_{(593\text{-}598\text{->}GSG)}$-*sfmturquoise2*^{ox}	Integration of pAI086 in MAB360
MAB515	CB15N Δ*dipM* P_{xyl}::P_{xyl}-*dipM* P_{van}::P_{van}-*dipM*$_{(K595A\ K597A)}$-*sfmturquoise2*^{ox}	Integration of pAI084 in MAB360

Supplementary Table 4. Plasmids used in this study.

Plasmid	Description	Reference/Construction
**Previously generated plasmids used in this study**		
pAM14	Integration plasmid carrying P_{xyl}-*venus-ftsN*, Kan^R	Möll et al., 2009
pAM68	Integration plasmid carrying *Pxyl-venus-ftsN*$_{(1-187)}$-*podJ*$_{(893-975)}$, Kan^R	Möll et al., 2009
pAM123	pNTPS138 derivative to generate an in-frame deletion of *amiC*, Kan^R	Möll et al., 2010
pAM210	Integration plasmid carrying P_{xyl}-*sdpA-mCherry*, Kan^R	Zielińska et al., 2017
pAZ14	Integration plasmid carrying P_{xyl}-*tat*^{SP}-*sdpB-mCherry*, Kan^R	Zielińska et al., 2017
pAZ37	Integration plasmid carrying P_{xyl}-*ldpF*, Kan^R	Zielińska et al., 2017
pET28(+)	Vector for overproduction of N-terminal fusions to His_6, Kan^R	Novagen
pMT814	pNTPS138 derivative to generate an in-frame deletion of *dipM*, Kan^R	Möll et al., 2010
pNPTS138	*sacB*-containing suicide vector used for double homologous recombination, Kan^R	M.R.K. Alley (unpublished)
pTB146	Vector for overproduction of N-terminal fusions to *His6-SUMO*, Amp^R	T. Bernhard (unpublished)
pVVENN-4	Integration plasmid to produce fusion proteins carrying an N-terminal Venus tag under the control of P_{van}, $Gent^R$	Thanbichler et al., 2007
pXCFPN-4	Integration plasmid to produce fusion proteins carrying an N-terminal eCFP tag under the control of P_{xyl}, $Gent^R$	Thanbichler et al., 2007
pXFLGC-2	Integration plasmid to produce fusion proteins carrying a C-terminal FLAG tag under the control of P_{xyl}, Kan^R	Thanbichler et al., 2007
pXGFPN-4	Integration plasmid to produce fusion proteins carrying an N-terminal eGFP tag under the control of P_{xyl}, $Gent^R$	Thanbichler et al., 2007
**Plasmids generated in this study**		
pAI001	pTB146 derivative for the overexpression of *his6-SUMO-dipM*$_{(459-609)}$, Amp^R	a) Amplification of *dipM* with oligos PFsumodipm and PRsumodipm.<br>b) Cloning of the fragment into pTB146 via *SapI* and *BamHI*.
pAI002	pXFLGC-2 derivative bearing *dipM-flag*, Kan^R	a) Amplification of *dipM* with oligos AM119 and AM121.<br>b) Cloning of the fragment into pXFLGC-2 via *NdeI* and *EcoRI*.
pAI003	pAI002 derivative, $Gent^R$	a) Restriction of both pAI002 and pXGFPC-4 with *NdeI* and *NheI*<br>b) Ligation of the fragment containing the *dipM-flag* fusion from pAI002 into the open pXGFPC-4
pAI013	pAI003 derivative bearing *sdpA-flag*, $Gent^R$	a) Amplification of *sdpA* with primers AM270 and AM329<br>b) Cloning of the fragment into pAI003 via *NdeI* and *EcoRI*
pAI014	pET28a(+) derivative bearing *sdpA*$_{(21-699)}$-*his6*, Kan^R	a) Amplification of *sdpA* with primers OAI022 and OAI023.<br>b) Cloning of the fragment into pET28a(+) via *EcoRI* and *NdeI*.
pAI015	pXCFPN-4 derivative bearing *sdpA*, $Gent^R$	a) Amplification of *sdpA* with primers OAI023 and AM329.<br>b) Cloning of the fragment into pXCFPN-4 via *NdeI* and *EcoRI*.
pAI016	pXFLGC-2 derivative bearing *ldpF-flag*, Kan^R	a) Amplification of *ldpF* with primers AM214 and AM215.<br>b) Cloning of the fragment into pXFLGC-2 via *NdeI* and *EcoRI*.
pAI018	pXFLGC-2 derivative bearing *crbA-flag*, Kan^R	a) Amplification of *crbA* with primers OAI026 and OAI027.<br>b) Cloning of the fragment into pXFLGC-2 via *NdeI* and *EcoRI*.
pAI019	pXFLGC-2 derivative bearing *crbA*, Kan^R	a) Amplification of *crbA* with primers OAI026 and OAI028.<br>b) Cloning of the fragment into pXFLGC-2 via *NdeI* and *EcoRI*.
pAI025	pTB146 derivative for the overexpression of *his6-SUMO-ldpF*$_{(25-351)}$, Amp^R	a) Amplification of *ldpF* with primers OAI038 and OAI039.<br>b) Cloning of the fragment into pTB146 via *SapI* and *BamHI*.
pAI026	pTB146 derivative for the overexpression of *his6-SUMO-sdpB*$_{(26-536)}$, Amp^R	a) Amplification of *sdpB* with primers OAI036 and OAI037.<br>b) Cloning of the fragment into pTB146 via *SapI* and *XmaI*
pAI029	pAI003 derivative bearing *amiC-flag*, $Gent^R$	a) Amplification of *amiC* with primers OAI047 and OAI048.<br>b) Cloning of the fragment into pAI003 via *NdeI* and *EcoRI*.

Supplementary Table 4. Plasmids used in this study (continued).

pAI036	pTB146 derivative for the overexpression of *his6-SUMO-ftsN*$_{(51\text{-}266)}$, Amp^R	a) Amplification of *ftsN* with primers OAI076 and OAI077.<br>b) Insertion of the fragment into pTB146 cut with *SacI* and *BamHI* via Gibson assembly.
pAI037	pTB146 derivative for the overexpression of *his6-SUMO-amiC*$_{(35\text{-}395)}$, Amp^R	a) Amplification of *amiC* with primers OAI074 and OAI075.<br>b) Insertion of the fragment into pTB146 cut with *SacI* and *BamHI* via Gibson assembly.
pAI038	pXFLGC-2 derivative that can be used to make C-terminal *sfmTurquoise2*^{ox} fusions under the control of P_{xyl}, Kan^R	a) Amplification of *sfmTurquoise2*^{ox} with a multiple cloning site with primers OAI031 OAI032.<br>b) Cloning of the fragment into pXFLGC-2 via *NdeI* and *NheI*.
pAI039	pAI038 derivative bearing *dipM-sfmTurquoise2*^{ox}, Kan^R	a) Amplification of *dipM* with primers AM119 and AM121.<br>b) Cloning of the fragment into pAI038 via *NdeI* and *EcoRI*.
pAI041	pVVENN-4 derivative bearing *dipM-sfmTurquoise2*^{ox}, Gent^R	a) Restriction of both pAI039 and pVVENN-4 with N*deI* and *NheI*.<br>b) Ligation of the fragment from pAI039 bearing *dipM-sfmTurquoise2*^{ox} into the pVVENN-4
pAI049	pTB146 derivative for the overexpression of *his6-SUMO-crbA*$_{(371\text{-}451)}$, Amp^R	a) Amplification of *crbA* with primers OAI109 and OAI110.<br>b) Insertion of the fragment into pTB146 cut with *SacI* and *BamHI* via Gibson assembly.
pAI063	pAI041 derivative with Kan^R	a) Restriction of both pAI041 and pXFLGC-2 with *NheI* and *NotI*.<br>b) Ligation of the fragment from pAI041 bearing *dipM-sfmTurquoise2*^{ox} into pXFLGC-2
pAI064	Derivative of pAI063 bearing *dipM*$_{(\Delta 34\text{-}458)}$-*sfmturquoise2*^{ox}, Kan^R	Site-directed mutagenesis of pAI063 with primers OAI181 and OAI182
pAI065	Derivative of pAI063 bearing *dipM*$_{(\Delta 34\text{-}390)}$-*sfmturquoise2*^{ox}, Kan^R	Site-directed mutagenesis of pAI063 with primers OAI183 and OAI184
pAI066	Derivative of pAI063 bearing *dipM*$_{(\Delta 123\text{-}458)}$-*sfmturquoise2*^{ox}, Kan^R	Site-directed mutagenesis of pAI063 with primers OAI185 and OAI186
pAI067	Derivative of pXGFPC-4 bearing *dipM-sfmTurquoise2*^{ox}, Gent^R	a) Restriction of both pAI063 and pXGFPC-4 with *NdeI* and *NheI*.<br>b) Ligation of the fragment from pAI063 bearing *dipM-sfmTurquoise2*^{ox} into pXGFPC-4
pAI068	Derivative of pXGFPC-4 bearing *dipM*$_{(\Delta 34\text{-}458)}$-*sfmTurquoise2*^{ox}, Gent^R	a) Restriction of both pAI064 and pXGFPC-4 with *NdeI* and *NheI*.<br>b) Ligation of the fragment from pAI064 bearing *dipM-sfmTurquoise2*^{ox} into pXGFPC-4
pAI072	Plasmid to generate an allele encoding the DipM$_{(\Delta 36\text{-}459)}$ variant at the native *dipM* locus, Kan^R	a) Amplification of the upstream and downstream regions from the *C. crescentus* chromosome using primers OAI190/OAI193 and OAI194/OAI195, respectively.<br>b) Insertion of both fragments into pNPTS138 cut with *BamHI* and *EcoRI* by Gibson assembly
pAI075	Derivative of pAI063 bearing *dipM*$_{(\Delta 390\text{-}609)}$-*sfmturquoise2*^{ox}, Kan^R	Site-directed mutagenesis of pAI063 with primers OAI199 and OA200
pAI077	Derivative of pAI063 bearing *dipM*$_{(\Delta 35\text{-}500)}$-*sfmturquoise2*^{ox}, Kan^R	Site-directed mutagenesis of pAI063 with primers OAI203 and OA204
pAI078	Derivative of pAI063 bearing *dipM*$_{(\Delta 35\text{-}478)}$-*sfmturquoise2*^{ox}, Kan^R	Site-directed mutagenesis of pAI063 with primers OAI205 and OA206
pAI079	Derivative of pAI063 bearing *dipM*$_{(\Delta 35\text{-}486)}$-*sfmturquoise2*^{ox}, Kan^R	Site-directed mutagenesis of pAI063 with primers OAI207 and OA208
pAI081	Derivative of pXGFPC-4 bearing *dipM*$_{(\Delta 35\text{-}500)}$-*sfmturquoise2*^{ox}, Gent^R	a) Restriction of both pAI077 and pXGFPC-4 with *NdeI* and *NheI*.<br>b) Ligation of the fragment from pAI063 bearing *dipM*$_{(\Delta 35\text{-}500)}$-*sfmTurquoise2*^{ox} into pXGFPC-4
pAI082	Derivative of pXGFPC-4 bearing *dipM*$_{(\Delta 35\text{-}478)}$-*sfmturquoise2*^{ox}, Gent^R	a) Restriction of both pAI078 and pXGFPC-4 with *NdeI* and *NheI*.<br>b) Ligation of the fragment from pAI063 bearing *dipM*$_{(\Delta 35\text{-}478)}$-*sfmTurquoise2*^{ox} into pXGFPC-4
pAI083	Derivative of pXGFPC-4 bearing *dipM*$_{(\Delta 35\text{-}486)}$-*sfmturquoise2*^{ox}, Gent^R	a) Restriction of both pAI079 and pXGFPC-4 with *NdeI* and *NheI*.<br>b) Ligation of the fragment from pAI063 bearing *dipM*$_{(\Delta 35\text{-}486)}$-*sfmTurquoise2*^{ox} into pXGFPC-4
pAI084	Derivative of pAI063 bearing *dipM*$_{(K595A,\ K598A)}$-*sfmturquoise2*^{ox}, Kan^R	Site-directed mutagenesis of pAI063 with primers OAI213 and OA214
pAI086	Derivative of pAI063 bearing *dipM*$_{(593\text{-}598\text{->}GSG)}$-*sfmturquoise2*^{ox}, Kan^R	Site-directed mutagenesis of pAI063 with primers OAI218 and OA219
pAI087	Derivative of pXGFPC-4 bearing *dipM*$_{(\Delta 390\text{-}609)}$-*sfmturquoise2*^{ox}, Gent^R	a) Restriction of both pAI075 and pXGFPC-4 with *NdeI* and *NheI*.<br>b) Ligation of the fragment from pAI075 bearing *dipM*$_{(\Delta 390\text{-}609)}$-*sfmTurquoise2*^{ox} into pXGFPC-4
pAI095	Derivative of pAI063 bearing *dipM*$_{(R589A)}$-*sfmturquoise2*^{ox}, Kan^R	Site-directed mutagenesis of pAI063 with primers OAI229 and OA230

Supplementary Table 3. Plasmids used in this study (continued).

pMAB150	pTB146 derivative for the overexpression of *his6-SUMO-ldtD$_{(26\text{-}502)}$*, Amp^R	a) Amplification of *ldtD* with primers MAB401 and MAB397.<br>b) Insertion of the fragment into pTB146 cut with *SapI* and *XhoI* via Gibson assembly.
pMAB194	pET28a(+) derivative encoding the R589A variant of $DipM_{(26\text{-}610)}$-His_6, Kan^R	a) Amplification of *dipM$_{(R589A)}$* from pAI095 with primers AM121 and AM124 and digestion of the product with *NcoI* and *EcoRI*<br>b) Ligation of the fragment into pET28a(+) cut with *NcoI* and *EcoRI*
pMAB195	Derivative of pAI063 bearing *dipM$_{(L537S)}$-sfmturquoise2^{ox}*, Kan^R	Site-directed mutagenesis of pAI063 with primers MAB507 and MAB508
pMAB198	Derivative of pAI063 bearing *dipM$_{(L539S)}$-sfmturquoise2^{ox}*, Kan^R	Site-directed mutagenesis of pAI063 with primers MAB509 and MAB510
pMAB203	Derivative of pAI063 bearing *dipM$_{(L537S\ L539S)}$-sfmturquoise2^{ox}*, Kan^R	Site-directed mutagenesis of pMAB195 with primers MAB515 and MAB516
pMAB206	pET28a(+) derivative encoding the L539S variant of $DipM_{(26\text{-}610)}$-His_6, Kan^R	a) Amplification of *dipM$_{(L539S)}$* from pMAB198 with primers AM121 and AM124 and digestion of the product with *NcoI* and *EcoRI*<br>b) Ligation of the fragment with pET28a(+) cut with *NcoI* and *EcoRI*
pMAB206	pET28a(+) derivative encoding the L537S L539S variant of $DipM_{(26\text{-}610)}$-His_6, Kan^R	a) Amplification of *dipM$_{(L537S\ L539S)}$* from pMAB203 with primers AM121 and AM124 and digestion of the product with *NcoI* and *EcoRI*<br>b) Ligation of the fragment with pET28a(+) cut with *NcoI* and *EcoRI*

Supplementary Table 5. Oligonucleotides used in this work.

Oligonucleotide name	Sequence
PFsumodipm	ggtggtagaagagcaggtcggacgatcatcgagaccgccgc
PRsumodipm	ggatcctcagcgcggcagcaccagcgccg
OAI022	ggcagccatatggctagcgccgctgacgcccagacgacgac
OAI023	tagaattcgcttaaggctgggcgctggccg
OAI025	ctgcagctagcttactctatcgcgactcctgtttgagccagc
OAI026	agacgaccatatggtgtggcggtgcggaaccgc
OAI027	gttcgaattctccggcttcagcacaatgcagggc
OAI028	gttcgaattctcctacggcttcagcacaatgcagggc
OAI036	ttggtggtagaagagcacacgccagcgggcttgagcccc
OAI037	cgagcccgggtgacctagggcagctgcgccatcaactggt
OAI038	ggtggtagaagagcacagcgcgccgacgccgccttcg
OAI039	aaggggatccggctttatcgcgactcctgtttgagccagcgc
OAI047	cggagaattcgaacaagacttgcgaagacccgaggag
OAI048	cgaccatatgcctgcatgcgtagaggtctcatcaatttcgct
OAI074	gtagaagagcagagctcggacccgccgcgcccgccg
OAI075	gctttgttagcagccggatccctaagacttgcgaagacccgagg
OAI076	gggctttgttagcagccggatcctcactttacgaagcaggatttgccgg
OAI077	ggtggtagaagagcagagctcatgatctatcgcgatggcgtgcgc
OAI109	ggtagaagagcagagctcgcgaagaagcctaagggcgaatg
OAI110	ggctttgttagcagccggatccctacggcttcagcacaatgcagg
OAI181	cttcacgccgggtcggacgatcatcgagaccgc
OAI182	gatcgtccgacccggcgtgaagcgctgaccc
OAI183	cgcttcacgccgcccgacggtttccgcgacaagg
OAI184	gaaaccgtcgggcggcgtgaagcgctgacccga
OAI185	gcaagccccagggtcggacgatcatcgagaccgc
OAI186	cgatgatcgtccgaccctggggcttgcccgcgac
OAI190	tctctgcaggatatctggatccggagatcggcaccggc
OAI193	cgtccgaccgaagttcggcgtgaagcgc
OAI194	cacggccgaagctagcgaattcccgctcgcgcgatgtacg
OAI195	gccgaacttcggtcggacgatcatcgagaccg
OAI199	cgcgctgcccgcgaattcgaacgttacgcgtcac
OAI200	tcgaattcgcgggcagcgcgatcttctgg
OAI203	gcttcacgccgcagcgcaacgacggcctcaatatccg
OAI204	gttgcgctgcggcgtgaagcgctgacccg
OAI205	ttcacgccgaacggcaagttcgcctggccgctg
OAI206	cgaacttgccgttcggcgtgaagcgctgacccga
OAI207	acgccgaacggcgacatcatctccagctttggcgt
OAI208	atgatgtcgccgttcggcgtgaagcgctgacccga
OAI213	tggcggacgcagccaagccggtcgatccggc
OAI214	gctgcgtccgccaccgtcggcgcgtagcgc
OAI218	ccgggatcgggtgccaagccggtcgatccggc
OAI219	cttggcacccgatcccggcgcgtagcgcatctcga
OAI227	cgcaacgccggcctcaatatccgcg
OAI228	ccggcgttgcgctggcccgtg
OAI229	gagatggcctacgcgccgacggtg
OAI230	gcgcgtaggccatctcgaagtgcagctgc
OAI231	tggtcgcgcacgccgacggctg
OAI232	gcgtgcgcgaccagcacgaggttgc
OAI233	ggccagcgcaacaagggcctcaatatccgc
OAI234	tattgaggcccttgttgcgctggcccgtgcc
AM119	ttttcatatgaggcagttgtggacgcaagcggc
AM121	tagaattcgcgcgcggcagcaccagcgcc
AM124	aaaccatggcgagccagtcgggtcagcgcttcacgcc
AM214	ttttcatatgtcccgctcgcgactcgtcttgg
AM215	tagaattcgctcgcgactcctgtttgagccagcgc
AM270	tagaattcgcaggctgggcgctggccgtctcggctg
AM329	ttttcatatggtttcaggaatgcgtcgctggc
MAB397	aggctcacagagaacagattggtggtcagtcgcagcgtccgcc
MAB400	tgcagtcacccgggctcgagtcagagtgcggcgatacgctg

Supplementary Table 5. Oligonucleotides used in this work (continued).

MAB507	aggtcccgacattcggcaactccgtgctggtcaagcacgccg
MAB508	cggcgtgcttgaccagcacggagttgccgaatgtcgggacct
MAB509	gacattcggcaacctcgtgtcggtcaagcacgccgacggct
MAB510	agccgtcggcgtgcttgaccgacacgaggttgccgaatgtc
MAB515	gacattcggcaactccgtgtcggtcaagcacgccgacggct
MAB516	agccgtcggcgtgcttgaccgacacggagttgccgaatgtc

Supplementary references

An, D.R., Im, H.N., Jang, J.Y., Kim, H.S., Kim, J., Yoon, H.J., Hesek, D., Lee, M., Mobashery, S., Kim, S.-J.*, et al.* (2016) Structural basis of the heterodimer formation between cell shape-determining proteins Csd1 and Csd2 from *Helicobacter pylori*. *PLoS One* **11**, e0164243.

Billini, M., Biboy, J., Kühn, J., Vollmer, W. & Thanbichler, M. (2019) A specialized MreB-dependent cell wall biosynthetic complex mediates the formation of stalk-specific peptidoglycan in *Caulobacter crescentus*. *PLoS Genet.* **15**, e1007897.

Cook, J., Baverstock, T.C., McAndrew, M.B.L., Stansfeld, P.J., Roper, D.I. & Crow, A. (2020) Insights into bacterial cell division from a structure of EnvC bound to the FtsX periplasmic domain. *Proc. Natl. Acad. Sci. USA* **117**, 28355-28365.

Evans, R., O'Neill, M., Pritzel, A., Antropova, N., Senior, A., Green, T., Žídek, A., Bates, R., Blackwell, S., Yim, J.*, et al.* (2022) Protein complex prediction with AlphaFold-Multimer. bioRxiv, DOI: 10.1101/2021.10.04.463034.

Evinger, M. & Agabian, N. (1977) Envelope-associated nucleoid from *Caulobacter crescentus* stalked and swarmer cells. *J. Bacteriol.* **132**, 294-301.

Grabowska, M., Jagielska, E., Czapinska, H., Bochtler, M. & Sabala, I. (2015) High resolution structure of an M23 peptidase with a substrate analogue. *Sci. Rep.* **5**, 14833.

Meisner, J., Maehigashi, T., André, I., Dunham, C.M. & Moran, C.P. (2012) Structure of the basal components of a bacterial transporter. *Proc. Natl. Acad. Sci. USA* **109**, 5446-5451.

Mistry, J., Chuguransky, S., Williams, L., Qureshi, M., Salazar, G.A., Sonnhammer, E.L.L., Tosatto, S.C.E., Paladin, L., Raj, S., Richardson, L.J.*, et al.* (2021) Pfam: The protein families database in 2021. *Nucleic Acids Res.* **49**, D412-D419.

Möll, A., Schlimpert, S., Briegel, A., Jensen, G.J. & Thanbichler, M. (2010) DipM, a new factor required for peptidoglycan remodelling during cell division in *Caulobacter crescentus*. *Mol. Microbio.l* **77**, 90-107.

Möll, A. & Thanbichler, M. (2009). FtsN-like proteins are conserved components of the cell division machinery in proteo-bacteria. *Mol. Microbiol.* **72**, 1037-1053.

Shin, J.-H., Sulpizio, A.G., Kelley, A., Alvarez, L., Murphy, S.G., Fan, L., Cava, F., Mao, Y., Saper, M.A. & Dörr, T. (2020) Structural basis of peptidoglycan endopeptidase regulation. *Proc. Natl. Acad. Sci. USA* **117**, 11692-11702.

Thanbichler, M., Iniesta, A.A. & Shapiro, L (2007) A comprehensive set of plasmids for vanillate- and xylose-inducible gene expression in *Caulobacter crescentus*. *Nucleic Acids Res.* **35**, e137.

Zielińska, A., Billini, M., Möll, A., Kremer, K., Briegel, A., Izquierdo Martinez, A., Jensen, G.J. & Thanbichler, M. (2017). LytM factors affect the recruitment of autolysins to the cell division site in *Caulobacter crescentus*. *Mol. Microbiol.* **106**, 419-438.
