# Supplementary material for: DipM controls multiple autolysins and mediates a regulatory feedback loop promoting cell constriction in Caulobacter crescentus
Source: Nat Commun. 2023 Jul 11;14:4095. doi: 10.1038/s41467-023-39783-w (PMC10336132; doi:10.1038/s41467-023-39783-w)
Supplement: Supplementary file 3 — Description of Additional Supplementary Files [file 41467_2023_39783_MOESM3_ESM.pdf]

Description of Additional Supplementary Files:

Supplementary Data 1. Proteins identified in the co-immunoprecipitation analysis. The tables give the annotations, Uniprot accession numbers, peptide counts, enrichment factors and p-values of all proteins copurifying with FLAG-tagged DipM, SdpA, CrbA, FtsN, AmiC and LdpF compared to the respective negative controls.
